# Account of Diseases in an Eastern District of London, from September 20, to October 20, 1804

**Published:** 1804-11-01

**Authors:** 


### *Account of Diseases in an Eastern District of London, from September* 20, *to October* 20, 1804.

ACUTE DISEASES.

| | |
|---|---|
| Typhus - - - - - - - | 2 |
| Ephemera - - - - - - | 3 |
| Dysenteria - - - - - - | 4 |
| Rheumatismus Acutus - | 2 |

CHRONIC DISEASES.

| | |
|---|---|
| Tussis - - - - - - - | 11 |
| Dyspnœa - - - - - - | 4 |
| Tussis cum Dyspnœa - | 9 |
| Pleurodyne - - - - - | 3 |
| Phthisis Pulmonalis - | 2 |
| Gastrodynia - - - - - | 6 |
| Dyspepsia - - - - - - | 7 |
| Hypochondriasis - - - | 4 |
| Hydrothorax - - - - - | 4 |
| Ascites - - - - - - - - | 3 |
| Diarrhœa - - - - - - | 17 |
| Chlorosis - - - - - - | 3 |
| Menorrhagia - - - | 2 |
| Vermes - - - - - - | 3 |
| Ischuria - - - - - - | 2 |
| Rheumatismus Chronicus | 17 |

PUERPERAL DISEASES.

| | |
|---|---|
| Menorrhagia Lochialis - | 5 |
| Dolores Post Partum - | 6 |
| Ephemera - - - - - - | 8 |
| Abscessus Mammæ - - | 2 |
| Hæmorrhois - - - - | 1 |

INFANTILE DISEASES.

| | |
|---|---|
| Diarrhœa - - - - - - | 12 |
| Aphthæ - - - - - - | 5 |
| Tinea - - - - - - - - | 2 |
| Vermes - - - - - - | 3 |
| Ophthalmia Purulenta - | 2 |

The diseases which usually occur at this season of the year prevail at present to a considerable degree. Complaints of the stomach and bowels are now very general.

Diarrhœa and dysentery have more frequently occurred than cholera. The first of these indeed may generally be considered as a salutary effort of the constitution to throw off something by which it is oppressed, and is very seldom productive of any serious consequence.

Though it may be necessary to restrain it within proper bounds, yet too early an interference is often injurious. Dysentery, though a distinct disease, has, sometimes, been so nearly connected with diarrhœa as to be mistaken for a continuance of the same disease. In the dysentery, though there is a frequent inclination to go to stool, the quantity discharged is very small, and consists chiefly of mucus or of of mucus and blood. Some degree of fever usually attends the disease; but it will sometimes continue, in a chronic kind of state, for a considerable time after the fever has subsided. The solution of this disease is generally promoted by the discharge of fæces; and those medicines which have been employed, perhaps with some other intention, have proved useful, in proportion as they have promoted the evacuation of fæculent matter which has been retained in the colon.

